# Target fluorescence *in-situ* hybridization (Target FISH) for plasma cell enrichment in myeloma

**DOI:** 10.1186/s13039-016-0263-7

**Published:** 2016-08-16

**Authors:** Edmond S. K. Ma, Candy L. N. Wang, Anthony T. C. Wong, Gigi Choy, Tsun Leung Chan

**Affiliations:** Division of Molecular Pathology, Department of Pathology, Hong Kong Sanatorium & Hospital, Clinical Pathology Laboratory, 1/F Li Shu Fan Block, 2 Village Road, Happy Valley, Hong Kong

**Keywords:** Plasma cell myeloma, Cytogenetics, FISH, Prognosis, Risk stratification, Molecular pathology, Cell sorting, Laboratory automation

## Abstract

**Background:**

Cytogenetic abnormalities are important prognostic markers in plasma cell myeloma (PCM) and detection is routinely performed by interphase fluorescence in-situ hybridization (FISH) with a panel of probes after enrichment of the plasma cells in the bone marrow specimen. Cell sorting by immunomagnetic beads and concurrent labeling of the cytoplasmic immunoglobulin are the usual enrichment methods. We present an alternative method of plasma cell enrichment termed Target FISH, which is an automated system that combines the images of May-Grünwald- Giemsa (MGG) staining and FISH study on the same plasma cell for analysis.

**Results:**

Our experience of Target FISH on 40 PCM patients was described. Briefly, plasma cells were MGG stained, image captured, de-stained, FISH probe hybridized and finally relocated for simultaneous analysis of morphology and FISH signal pattern. The FISH probe panel was *TP53*/CEP17, t(4;14) *IGH*/*FGFR3*, t(14;16) *IGH*/*MAF* and *CKS1B*(1q21)/*CDKN2C*(P18). Gain of 1q21 was the most common abnormality detected in 18 patients (45 %), to be followed by t(4;14) *IGH*/*FGFR3* detected in 11 patients (27.5 %). Of note, 10 patients showed coexistence of both t(4;14) and 1q21 gain. Two patients showed del(17p)/*TP53*, one in association with t(4;14) and 1q gain while the other was stand alone. None of this patient cohort showed t(14;16) *IGH*/*MAF*. Using the critical binomial function, the normal cutoff FISH positive value for del(17p)/*TP53* was 3.4 %, t(4;14) *IGH*/*FGFR3* was 6.8 %, t(14;16) *IGH*/*MAF* was 5.6 % and +1q21 was 5.7 %.

**Conclusions:**

The equipment cost notwithstanding, when compared with cell sorting, the total reagent cost was around 10 % lower in Target FISH. The total processing time was longer for Target FISH but manual fluorescence microscopy was no longer necessary. The main advantage of Target FISH was the complete certainty that the cytogenetic abnormality was detected in the cells of interest, and hence a more stringent analytical cutoff value might be considered. Optimization of the cell collection and slide preparation process upfront was required to accrue adequate target cells on each slide for analysis. Our experience suggested that Target FISH was applicable as a routine method of plasma cell enrichment in clinical diagnostic laboratories.

## Background

The detection of cytogenetic abnormalities in PCM is clinically important as a prognostic marker to risk stratify patients [1]. While conventional metaphase chromosome study detects cytogenetic abnormalities in only one third of PCM patients, interphase FISH improves the detection frequency to around 90 % of patients [2]. The reasons for the discrepancy are attributable to low plasma cell percentage in the bone marrow, low proliferative index of the plasma cells and hence outgrown by granulocytic precursors, and that chromosome translocations such as t(4;14)(p16;q32) may be morphologically cryptic or co-exist with other complex changes and escape detection by the less experienced cytogeneticist.

It is not recommended to perform interphase FISH directly on the PCM bone marrow due to often low plasma cell percentage and admixture by other hemopoietic cells. The plasma cells should be selected either by immunomagnetic beads or flow cytometry based plasma cell sorting or concurrent labeling of the cytoplasmic immunoglobulin (cIg) light chain to allow unambiguous detection of cytogenetic abnormalities in the neoplastic plasma cell population[3, 4]. The enrichment techniques of cell sorting [5] or cIg-FISH [6] however are labor, time and cost intensive. Hence the incorporation of these methods into the routine workflow of a diagnostic cytogenetics laboratory may be challenging. An alternative method of plasma cell enrichment termed Target FISH based on sequential MGG stain to identify plasma cell populations to be followed by FISH analysis was previously reported [7]. Herein, we present an automated Target FISH system for use in routine molecular diagnostics.

## Results and discussion

We reported the experience on the initial 40 PCM patients for whom Target FISH was performed (Table 1). These patients were accrued in a 12-month period from April 2015 to March 2016. The patients comprised 20 males and females each at a median age of 61 years (range 47–89 years). The median percentage of plasma cells in the bone marrow was 29 % (range 12–100 %). Monoclonal gammopathy of undetermined significance (MGUS) were excluded. In the same time period, two samples in which FISH was failed (due to heavy background fluorescence and aged sample respectively) and one inadequate sample were also excluded. Conventional cytogenetic study results were available in only 10 patients (no. 2, 7, 9, 13, 17, 21, 22, 25, 33 and 35) for correlation (Table 1).

**Table 1:** Summary of Target FISH results on 40 patients

No.	Sex/Age	PC%^a^	BioView^b^	Del(17p)/*TP53*	t(4;14)	t(14;16)	+1q21	Others
1	M/52	26 %	14–16 % (10323–15875)	Neg (230)^c^	38 % + (74)	Neg (201)	30 % + (180)	
2	F/80	15 %	3–4 % (9605–13508)	Neg (89)	Neg (61)	Neg (71)	Neg (71)	Normal cytogenetics
3	M/69	12 %	3–4 % (8880–11094)	Neg (84)	Neg (46)^g^	Neg (40)^g^	Neg (62)	
4	M/81	43 %	No record	Neg (253)	Neg (188)	Neg (175)	Neg (200)	5/9/15 60 % + (235)
5	M/64	15 %	58–64 % (11912–17007)	Neg (159)	Neg (153)	Neg (156)^g^	Neg (155)	5/9/15 Neg (84)
6	M/70	17 %	4–7 % (7132–7616)	Neg (63)	Neg (53)^g^	Neg (51)^g^	80 % + (53)	5/9/15 Neg (61)
7	F/52	35 %	8–12 % (7750–8981)	Neg (159)	90 % + (112)^g^	Neg (102)^g^	65 % + (167)	Cytogenetics^d^: hypodiploid 78.5 %, hyperdiploid 7 %, normal 14.5 %
8	F/72	13 %	42–55 % (11829–14627)	Neg (32)	Neg (35)	Neg (71)	Neg (54)	
9	F/71	17 %	7–9 % (8667–11174)	Neg (82)	70 % + (54)	Neg (37)	50 % + (65)	Normal cytogenetics
10	M/61	65 %	17–24 % (10289–13997)	Neg (135)	Neg (94)^g^	Neg (98)^g^	90 % + (145)	
11	M/54	15 %	15–22 % (12898–19268)	Neg (141)	80 % + (87)	Neg (84)	Neg (113)	5/9/15 Neg (171)
12	M/47	100 %	50–59 % (10424–12419)	50 % + (132)	Neg (144)^g^	Neg (107)^g^	Neg (156)	
13	M/59	60 %	10–12 % (9514–13412)	Neg (154)	Neg (133)^g^	Neg (128)^g^	Neg (140)	t(11;14) 90 %+; normal cytogenetics
14	M/81	22 %	50–52 % (10110–15964)	Neg (140)	60 % + (78)^g^	Neg (111)^g^	85 % + (172)	5/9/15 Neg (101)
15	F/67	45 %	21–27 % (6066–7366)	Neg (163)	90 % + (145)	Neg (136)	85 % + (183)	5/9/15 Neg (157)
16	M/58	No record	8–9 % (6666–8923)	Neg (250)	Neg (192)^g^	Neg (188)^g^	Neg (224)	t(11;14) 25 % + (228)
17	F/70	51 %	13–17 % (7469–8551)	Neg (183)	Neg (137)^g^	Neg (118)^g^	85 % + (162)	Normal cytogenetics
18	F/53	23 %	10 % (2719–4195)	Neg (198)	Neg (146)	Neg (80)	Neg (106)	5/9/15 Neg (87)
19	M/76	70 %	6–7 % (4145–5686)	Neg (108)	Neg (89)	Neg (102)	Neg (101)	IgH Neg (71)
20	M/61	49 %	17–19 % (4210–5352)	Neg (170)	Neg (126)	Neg (119)	85 % + (172)	
21	M/61	20 %	24–29 % (9895–11736)	Neg (145)	Neg (122)	Neg (87)	Neg (142)	Normal cytogenetics
22	F/48	59 %	6–9 % (6942–7583)	40 % + (172)	65 % + (110)	Neg (111)	55 % + (154)	Normal cytogenetics
23	F/56	100 %	3–8 % (4400–6203)	Neg (123)	Neg (149)^g^	Neg (118)	65 % + (158)	
24	F/55	84 %	3–6 % (9304–10362)	Neg (141)	Neg (148)	Neg (107)	Neg (82)	t(11;14) 85 % + (121)
25	M/52	74 %	11–23 % (10024–12394)	Neg (158)	85 % + (100)	Neg (135)^g^	90 % + (162)	Cytogenetics^e^: hypodiploid clone, der(4)t(1;4)(q21;p16) and add(7p)
26	F/56	35 %	11–17 % (10247–12375)	Neg (100)	Neg (166)	Neg (140)^g^	Neg (117)	
27	F/55	73 %	10–17 % (3419–5581)	Neg (186)	Neg (167)	Neg (142)	Neg (171)	
28	F/57	15 %	6–8 % (5191–6330)	Neg (102)	Neg (122)	Neg (102)	Neg (100)	
29	F/69	16 %	25–32 % (7923–12371)	Neg (172)	Neg (143)^g^	Neg (104)^g^	Neg (173)	
30	M/58	19 %	3–5 % (6385–8924)	Neg (213)	Neg (169)	Neg (154)	Neg (208)	
31	F/66	29 %	7–11 % (7383–9153)	Neg (149)	70 % + (73)	Neg (108)^g^	80 % + (127)	
32	F/56	15 %	7–12 % (11256–13079)	Neg (146)	Neg (117)^g^	Neg (93)	Neg (150)	
33	M/64	13 %	4–6 % (11082–12940)	Neg (118)	Neg (100)	Neg (98)	Neg (101)	t(11;14) 40 % + (149); cytogenetics^f^: loss of Y chromosome
34	F/67	21 %	6–13 % ^g^11872–14598)	Neg (159)	70 % + (104)	Neg (118)^g^	95 % + (136)	
35	F/89	12 %	3–5 % (11793–14289)	Neg (121)	Neg (134)	Neg (102)	Neg (102)	Normal cytogenetics
36	F/50	56 %	14–17 % (10614–13489)	Neg (140)	Neg (143)	Neg (111)	60 % + (137)	
37	M/60	50 %	24–27 % (10633–13622)	Neg (165)	Neg (121)	Neg (117)	83 % + (143)	
38	M/79	20 %	6–10 % (13896–15969)	Neg (125)	Neg (96)	Neg (121)^g^	80 % + (144)	80 % trisomy *TP53*
39	F/67	53 %	32–38 % (13807–17047)	Neg (162)	63 % + (123)	Neg (103)	63 % + (104)	
40	M/69	57 %	6–13 % (12778–15272)	Neg (158)	Neg (127)	Neg (148)	Neg (158)	

*Key:*

^a^The PC% refers to the plasma cell percentage in the bone marrow aspirate as enumerated on microscopic examination

^b^The BioView data refers to the percentage of cells in the plasma cell category as recognized by the automated image capture and analysis system before manual re-classification. The number in the parentheses refers to the total number of cells analyzed by the automated image capture and analysis system on the slide. Both are reported as a range of figures obtained from the 4 slides

^c^The number in parenthesis is the actual number of re-classified plasma cells with optimal FISH signals on which the positive or negative result is based. This figure is also indicated on the pathology report for each FISH probe

^d^26 ~ 45,XX,t(4;14)(p16;q32)[33]/48 ~ 159,XX[3]/46,XX[6]

^e^43 ~ 44,XY,+3[2],der(4)t(1;4)(q21;p16)[2],add(7)(p13)[2],+7 ~ 8mar[2][cp2]/46,XY[4]

^f^45,X,-Y[3]/46,XY[15]

^g^Denotes the incidental finding of secondary abnormalities i.e. monosomy of deletion of chromosomes 4, 14 or 16; trisomy or amplification of chromosomes 4, 14 or 16

Gain of 1q21 was the most common abnormality detected in 18 patients (45 %), which was consistent with the high prevalence of this cytogenetic abnormality reported in the literature [8–10]. The t(4;14) *IGH*/*FGFR3* was detected in 11 patients (27.5 %). Of note, 10 patients showed coexistence of both t(4;14) and 1q21 gain. Again this observation was consistent with the much overlap between adverse IgH translocations and 1q21 gain as reported by the UK MRC Myeloma IX Trial [11]. Two patients showed del(17p)/*TP53*, one in association with t(4;14) and 1q gain while the other was stand alone. None of this patient cohort showed t(14;16) *IGH*/*MAF*. However 4 patients tested positive for t(11;14) *IGH*/*CCND1*, which was performed selectively when the myeloma cells showed small lymphoplasmacytic morphology and expression of CD20 [12]. Incidental finding of secondary abnormalities i.e. monosomy or deletion of chromosomes 4, 14 or 16 or trisomy or amplification of chromosomes 4, 14 or 16 detected by t(4;14) *IGH*/*FGFR3* and/or t(14;16) *IGH*/*MAF* FISH probes were encountered in 17 patients.

The normal cutoff value to define FISH positivity for each probe was determined in the laboratory by studying a cohort of 6 normal control samples together with the negative results in the patient samples for the FISH probes del(17p)/*TP53* (n = 38), t(4;14) *IGH*/*FGFR3* (n = 29), t(14;16) *IGH*/*MAF* (n = 40) and +1q21 (n = 22). The 6 normal controls were patients with reactive plasmacytosis proven by absence of paraprotein and lack of light chain restriction on the plasma cells. This normal control cohort consisted of 2 male patients and 4 female patients with an age range of 41–81 years. The diagnoses were metastatic colon cancer, adult Still’s disease, anaplastic large cell lymphoma, incidental finding of increased globulins on checkup, primary osseous diffuse large B-cell lymphoma and primary hyperparathyroidism. The range of bone marrow plasma cells was 6–8 %. The definition of false positive signal pattern was less than 2 red signals for *TP53*/CEP17, any yellow fusion signal for t(4;14) *IGH*/*FGFR3* or t(14;16) *IGH*/*MAF*, and more than 2 red signals for chromosome 1q *CKS1B*/*CDKN2C*. The critical binomial function of the Microsoft Excel spreadsheet was used to determine the 95 % confidence limit of normal cutoff value [13]. The normal cutoff value for *TP53*/CEP17 was 3.4 %, t(4;14) *IGH*/*FGFR3* was 6.8 %, t(14;16) *IGH*/*MAF* was 5.6 % and chromosome 1q *CKS1B*/*CDKN2C* was 5.7 %. However, for clinical reporting, conservative cutoff levels of 10 % for fusions and 20 % for numerical abnormalities were generally recommended [4]. In the literature, other cutoff values were quoted, for example 30 % for chromosome 1q gain [14] and 60 % for del(17p) [15]. Notwithstanding the analytical validation, the clinical significance of the cutoff value should also be considered. For example, a study on bortezomib-based chemotherapy in PCM patients according to the copy number of 1q21 showed that 20 % of involved plasma cells or more had no significant difference on survival, indirectly confirming the validity of 20 % rather than a higher cutoff in defining 1q gain [16]. Similarly another FISH study showed that 50 % for 17p deletion and 20 % for 1q gain were the optimal cutoff values associated with the greatest survival difference for predicting poor clinical outcome [17].

A caveat of Target FISH was that although thousands or ten thousands of cells were captured by the system on each slide, the percentage categorized as plasma cells by the machine before manual visualization and re-classification was uneven and often discordant with the percentage determined by microscopic examination of the bone marrow aspirate smears. Notably, a lower BioView percentage was observed in 32 patients (80 %) in this cohort (Table 1). This resulted in calling the FISH result based on less than the target of at least 100 analyzable cells for each probe. This scenario was seen consistently across all probes in only 5 patients. Further optimization of the density gradient centrifugation and cytospin preparation were required to prevent this apparent “cell loss” in the Target FISH experiments. Following on this, Target FISH might not be applicable to the study of MGUS in which the plasma cell percentage was low, in contrast to cell sorting in which a larger volume of bone marrow blood could be used if available.

Before the implementation of Target FISH, plasma cell enrichment was performed by cell sorting with CD138 immunomagnetic beads (Miltenyi Biotec GmbH, Germany) on 253 patients and this cohort did not include the 40 patients for whom Target FISH was performed. The cohort consisted of 161 male and 92 female patients at a median age of 61 years (range: 38–88 years). The median bone marrow plasma cell percentage was 42 % and cell sorting was only performed in the 140 patients (55 %) with less than 50 % plasma cells in the bone marrow. The purity after cell sorting was checked by cytospin preparation. The volume of bone marrow sample was 1–3 mLs and comparable with Target FISH. Fluorescence microscopy was performed by two observers who analyzed at least 100 cells each. Positive cutoff levels of 10 % for fusions and 20 % for numerical abnormalities were adopted for reporting. For comparison with Target FISH, the frequency of cytogenetic abnormalities was 41.9 % for +1q21, 16.6 % for t(4;14) *IGH*/*FGFR3*, 0.9 % for t(14;16) *IGH*/*MAF* and 7.5 % for del(17p)/*TP53*.

The workflow and cost of Target FISH was compared with cell sorting by immunomagnetic beads (Table 2). Unfortunately our laboratory had no experience with cIg-FISH which precluded any comparison. The Target FISH was a 3-day procedure but for urgent cases the first 2 days could be combined into one day to stop at the probe hybridization step overnight to obtain the FISH results the next day. The total reagent cost of Target FISH was around 10 % less expensive than cell sorting because there was no need to employ the immunomagnetic beads. The equipment cost however had to be taken into consideration. The total processing time of Target FISH however was longer than cell sorting. This notwithstanding, the 3 h (4 slides at 45 min each) of BioView slide scanning on day 3 were performed automatically by the machine after the cell matching was verified, instead of the tedious 2-h manual examination under fluorescence microscopy in the dark room. Moreover, the reporting procedure was facilitated since all the captured images could be reviewed on the computer monitor. Under manual examination only the representative images were captured and one would resort to repeat fluorescence microscopy for counterchecking results. The manpower requirement was equivalent for both Target FISH and cell sorting.

**Table 2:** Comparison between cell soring by immunomagnetic beads and Target FISH

Cell sorting by immunomagnetic beads				Target FISH			
Procedure		Time (hours)	Reagent Cost (USD)	Procedure		Time (hours)	Reagent Cost (USD)
Day 1	Plasma cell sorting by MACS whole blood CD138 μ-beads	1.5	60	Day 1	Plasma cell enrichment by Ficoll	1.5	12
					Cytospin slides (10 slides per sample)	0.5	29
	Cytospin slides (10 slides per sample)	0.5	29		MGG staining	0.5	9
					BioView scan and review	2.0	0
Day 2	Lysis of red blood cells on slide	0.5	2	Day 2	De-staining of MGG	1.5	2
	FISH hybridization	3.0	236		FISH hybridization	3.0	236
	Probe hybridization	Overnight	0		Probe hybridization	Overnight	0
Day 3	Post-hybridization	1.0	13	Day 3	Post-hybridization	1.0	13
	Manual slide examination	2.0	0		BioView slide scan	3–4	0
	Review results, data analysis and reporting	2.0	0		Review results, data analysis and reporting	2.0	0
Total		10.5	340	Total		15–16	301

## Conclusion

Correlation of plasma cell morphology with FISH by manual means [18] was not a new concept. Neither was automated image analysis an entirely new concept for the purpose of plasma cell identification in myeloma FISH [19]. Consistent with a previous report in the literature [7], the Target FISH system presented in our study was capable of combining the two processes in a fully automated fashion for relocation of plasma cells to allow the analysis of the MGG image and FISH signals of the same plasma cell. In this way, it was 100 % ensured that the cytogenetic abnormality - if any was detected - was found in the cells of interest. Target FISH saved the cost on reagent, obviated the need for tedious manual fluorescence microscopy and enriched the sample to completely focus on the analysis of plasma cells. We reckoned that Target FISH was applicable as an attractive alternative to cell sorting and cIg-FISH as a method of plasma cell enrichment for detection of PCM cytogenetic abnormalities in clinical molecular diagnostic laboratories.

## Methods

### Bone marrow preparation for morphology

Mononuclear cells in the bone marrow were separated by density gradient centrifugation to prepare cytospin slides for staining and morphological examination by BioView system (Abbott Molecular, Des Plaines, IL). Around 2 mL of bone marrow blood in EDTA was processed. Briefly, bone marrow was diluted 1:4 with wash buffer and added slowly to separation reagent prepared from 1 volume of Histopaque 1.077 (Sigma) mixed with 3 volumes of Histopaque 1.119. The mixture was centrifuged at 500 g for 30 min and decelerated slowly for 30 min to zero. Care was taken not to disturb the different layer when removing the tube from the centrifuge. The mononuclear cell layer at the interface between the plasma layer above and the Ficoll-hypaque layer below was carefully pipetted off to another tube and washed twice. The cell pellet was re-suspended in morphology preserver consisting of Fetal Bovine Serum in Ham’s F-10 Nutrient Mix (Life Technologies).

### Preparation of cytospin slides and MGG Staining

The cytospin centrifuge produced single-layer cell preparations that also flattened the cells for optimal cytoplasmic and nuclear presentation required for morphology analysis. 10–20 mLs of cell pellet were added to 2–3 mLs of Morphology Preserver to create the cell suspension. Up to 200 μLs of this suspension was loaded to a cuvette and centrifuged at 1500 rpm for 5 min. The slide was then carefully extracted from the cuvette and allowed to dry after marking the area around the cells. The cell density was checked under light microscopy by a 20x dry objective to ensure single layer of cells that showed minimal cellular overlap and no clustering. High concentration caused the cells to overlap hence difficult to scan and classify, and low concentration reduced the analyzable cell number that adversely affected the test result. The concentration of the suspension should be adjusted by dilution or addition of more cell pellet as deemed necessary. At least 8 cytospin slides were made, 4 for the 4-probe myeloma FISH panel used in our laboratory and 4 for backup. Slides could be stored at −20 °C for up to 2 months until staining.

The cytospin slides were stained successively in May-Grünwald stain for 2 min and in 5 % Giemsa stain for 8 min. After drying, the slides were checked under light microscopy to confirm the appropriate staining quality for BioView scanning. Freshly prepared MGG stains were prepared each time to ensure optimal staining quality for plasma cell image recognition by BioView.

### Automated scanning of the MGG-stained slides by BioView

**Fig. 1:**
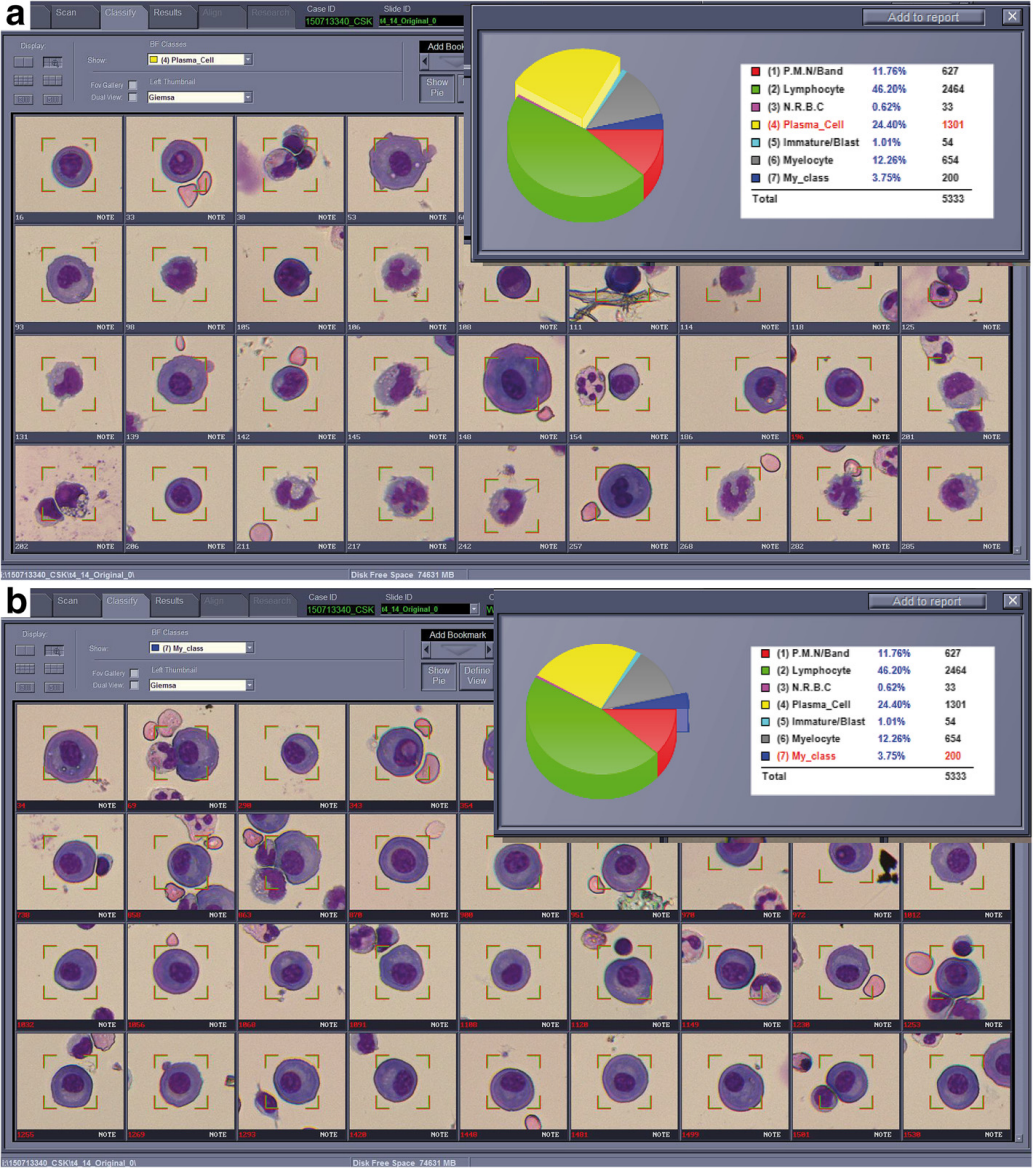
**a** From 5333 cells that were captured on this particular MGG stained slide, the image capture and analysis system automatically assigned 1301 cells to the plasma cell category. **b** The plasma cell category was manually reviewed to reclassify *bona fide* or clearly abnormal plasma cells into My_class category, which contained 200 cells in this case

The slide was loaded onto the stage of BioView for scanning and time required was around 15–20 min per slide depending on the cell quantity. After scanning, the plasma cell category as automatically selected by the machine was reviewed and *bona fide* plasma cells were reclassified into ‘my_class’ (Fig. 1). The aim was to obtain 200 plasma cells per slide for FISH to allow for hybridization failure since the reporting target was at least 100 plasma cells with clear FISH signals. If insufficient plasma cells were obtained, other categories were reviewed to move plasma cells into ‘my_class’ category.

### MGG de-staining and interphase FISH procedure

De-staining was performed by immersing slide in ice-cold (−20 °C) Carnoy’s solution (methanol: acetic acid 3:1) for 2 min at room temperature, and then moved to be immersed in another jar for 1 h. The slide was rinsed in 1x phosphate buffered saline for 5 min to be ready for FISH.

The pre-hybridization, probe hybridization and post-hybridization procedures were performed in accordance with standard leukemia lymphoma FISH protocols [20, 21]. Four FISH probes constituted the myeloma panel in our laboratory based on recommendations of the International Myeloma Working Group [1, 3, 22], namely Vysis *TP53*/CEP17 FISH probe kit (5 N56-20), Vysis *IGH*/*FGFR3* DF FISH probe kit (1 N69-20), Vysis *IGH*/*MAF* DF FISH probe kit (5 N32-20) from Abbott Molecular, and *CKS1B*/*CDKN2C*(P18) Amplification/Deletion probe kit (LPH 039) from Cytocell (Cambridge, UK). Other FISH probes such as cyclin D1 gene amplification [23] were not routinely applied. However the *IGH*/*CCND1* probe would be used when t(11;14) myeloma was suspected. Probe hybridization was performed on either Vysis HYBrite or ThermoBrite (Abbott Molecular).

### Scanning FISH slides by BioView

The slides were mounted on the stage and immersion oil was applied manually. Through the computer terminal, the corresponding case and slide number were documented, and the appropriate scanning task and probe name were selected. The images of the MGG-stained plasma cells were retrieved from the ‘re-visit my_class’ of the scan program and a dual-view screen was opened, in which a live image of the slide under fluorescence microscopy was displayed on the left side and the bright field image of the plasma cells on the right side, thus allowing direct matching of the FISH signal and morphology. The BioView captured images from 13 focal planes spaced 0.65 μm apart for each cell to minimize chance juxtaposition of FISH signals as a cause of false positive fusion signal pattern. Once a relocated plasma cell with clear FISH signals was identified from the live image, the bright field image previous captured was checked for accuracy of localization based on size, shape and surrounding cells. If a match was verified, both images were centered and the process was finished for that particular plasma cell. The process was then repeated for as many cells on whatever number of slides as required by the laboratory. We aimed to select at least 100 cells per slide with clear FISH signals, and 4 slides were used per patient for a myeloma panel of 4 probes. When all the slides for the case were properly configured, the slides were scanned automatically at around 45 min per slide.

**Fig. 2:**
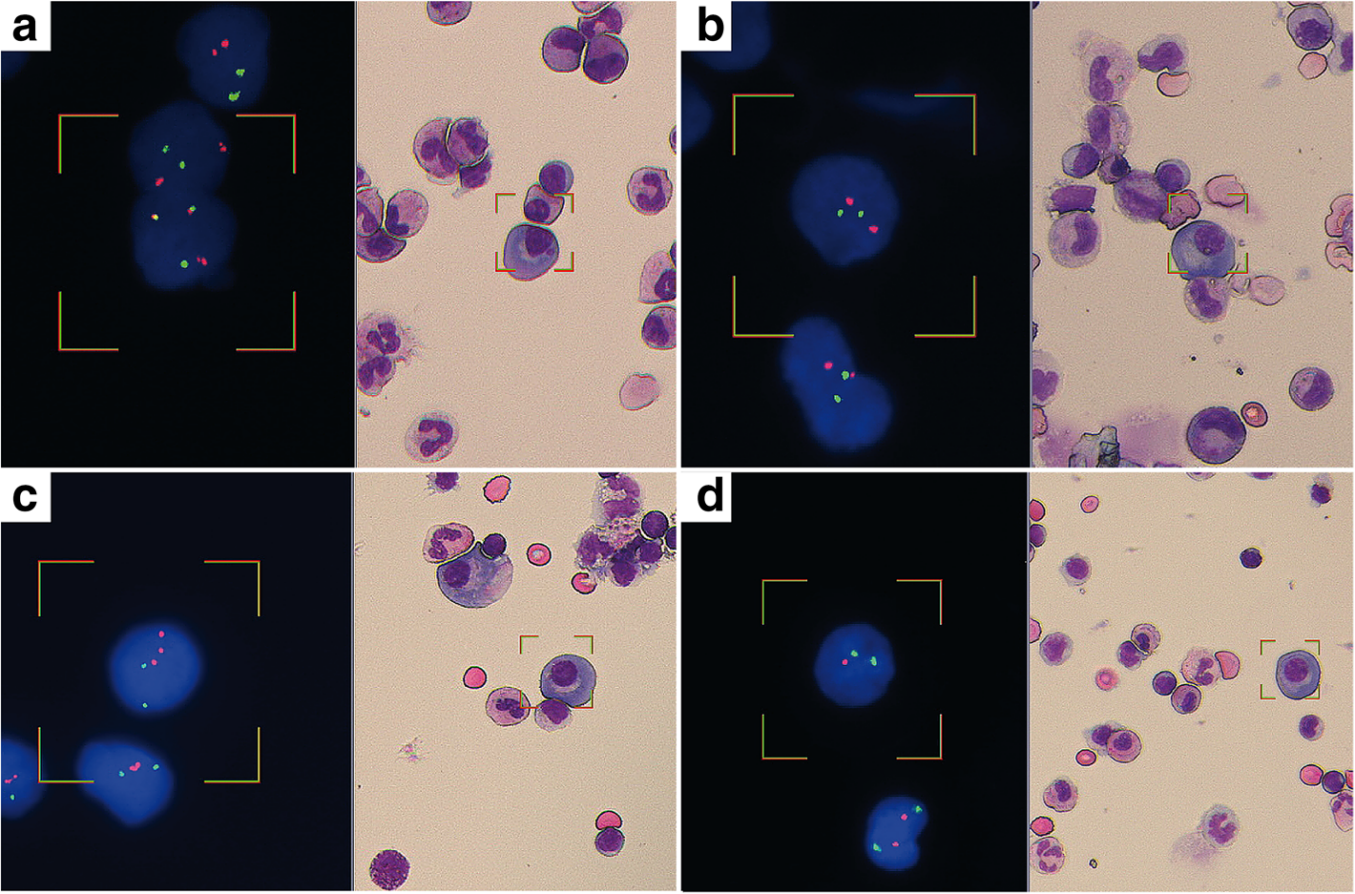
Images of MGG stained (*right*) and FISH probe hybridized (*left*) plasma cells. **a** t(4;14) *IGH*/*FGFR3* probe, showing the 2F1O1G signal pattern on a positive plasma cell. **b** t(14;16) *IGH*/*MAF* probe, showing the 2O2G signal pattern on a negative plasma cell. **c** *CKS1B*/*CDKN2C*(P18) probe, showing 3O2G signal pattern on the plasma cell positive for +1q21. **d** *TP53*/CEP17 probe, showing 1O2G signal pattern on the plasma cell positive for del(17p)/*TP53*

When scanning was completed, the results were manually visualized from the BioView software. A statistically summary of the FISH signal patterns of the selected plasma cells of each particular probe was displayed. Moreover, a composite image of FISH on the left side and MGG-stained picture of the same plasma cell on the right side could be prepared for inclusion in the pathology report (Fig. 2).

## Abbreviations

FISH, fluorescence in-situ hybridization; MACS, magnetic-activated cell sorting; MGG, May-Grünwald-Giemsa; PCM, plasma cell myeloma; SD, standard deviation
